# Supplementary material for: Stacking Model‐Based Classifiers for Dealing With Multiple Sets of Noisy Labels
Source: Biom J. 2025 Mar 12;67(2):e70042. doi: 10.1002/bimj.70042 (PMC11898607; doi:10.1002/bimj.70042)
Supplement: Supplementary file 1 — Supporting Information [file BIMJ-67-e70042-s001.zip › density-based-ensemble-model-main/competitors_real_data/netlab-master/netlab-master/help/gmmem.htm]

Netlab Reference Manual gmmem


# gmmem

## Purpose

EM algorithm for Gaussian mixture model.

## Synopsis

```
[mix, options, errlog] = gmmem(mix, x, options)
```

## Description

`[mix, options, errlog] = gmmem(mix, x, options)` uses the Expectation
Maximization algorithm of Dempster et al. to estimate the parameters of
a Gaussian mixture model defined by a data structure `mix`.
The matrix `x` represents the data whose expectation
is maximized, with each row corresponding to a vector.
The optional parameters have the following interpretations.

`options(1)` is set to 1 to display error values; also logs error
values in the return argument `errlog`.
If `options(1)` is set to 0,
then only warning messages are displayed. If `options(1)` is -1,
then nothing is displayed.

`options(3)` is a measure of the absolute precision required of the error
function at the solution. If the change in log likelihood between two steps of
the EM algorithm is less than this value, then the function terminates.

`options(5)` is set to 1 if a covariance matrix is reset to its
original value when any of its singular values are too small (less
than MIN\_COVAR which has the value eps).
With the default value of 0 no action is taken.

`options(14)` is the maximum number of iterations; default 100.

The optional return value `options` contains the final error value
(i.e. data log likelihood) in
`options(8)`.

## Examples

The following code fragment sets up a Gaussian mixture model, initialises
the parameters from the data, sets the options and trains the model.

```
mix = gmm(inputdim, ncentres, 'full');

options = foptions;
options(14) = 5;
mix = gmminit(mix, data, options);

options(1)  = 1;		% Prints out error values.
options(14) = 30;		% Max. number of iterations.

mix = gmmem(mix, data, options);
```

## See Also

`gmm`, `gmminit`

---

**Pages:**
Index


---

Copyright (c) Ian T Nabney (1996-9)
